# Erythrosine–Dialdehyde Cellulose Nanocrystal Coatings for Antibacterial Paper Packaging

**DOI:** 10.3390/polym16070960

**Published:** 2024-04-01

**Authors:** Shih-Chen Shi, Sing-Wei Ouyang, Dieter Rahmadiawan

**Affiliations:** 1 Department of Mechanical Engineering, National Cheng Kung University, No.1, University Road, Tainan 70101, Taiwan; n16074899@mail.ncku.edu.tw (S.-W.O.); n18127046@gs.ncku.edu.tw (D.R.); 2 Department of Mechanical Engineering, Universitas Negeri Padang, Padang 25173, Indonesia

**Keywords:** photodynamic antibacterial, cellulose, biopolymer, circular economy, biopolymer

## Abstract

Though paper is an environmentally friendly alternative to plastic as a packaging material, it lacks antibacterial properties, and some papers have a low resistance to oil or water. In this study, a multifunctional paper-coating material was developed to reduce the use of plastic packaging and enhance paper performance. Natural cellulose nanocrystals (CNCs) with excellent properties were used as the base material for the coating. The CNCs were functionalized into dialdehyde CNCs (DACNCs) through periodate oxidation. The DACNCs were subsequently complexed using erythrosine as a photosensitizer to form an erythrosine–CNC composite (Ery-DACNCs) with photodynamic inactivation. The Ery-DACNCs achieved inactivations above 90% after 30 min of green light irradiation and above 85% after 60 min of white light irradiation (to simulate real-world lighting conditions), indicating photodynamic inactivation effects. The optimal parameters for a layer-by-layer dip coating of kraft paper with Ery-DACNCs were 4.5-wt% Ery-DACNCs and 15 coating layers. Compared to non-coated kraft paper and polyethylene-coated paper, the Ery-DACNC-coated paper exhibited enhanced mechanical properties (an increase of 28% in bursting strength). More than 90% of the bacteria were inactivated after 40 min of green light irradiation, and more than 80% were inactivated after 60 min of white light irradiation.

## 1. Introduction

The food industry is under increasing pressure to reduce its reliance on plastic packaging, which is derived from non-renewable resources and contributes significantly to environmental pollution and landfill waste [1]. It also has limited protection against microbial contamination, which can compromise food safety and reduce shelf life. Therefore, consumers have become more inclined to reduce environmental pollution and the use of plastic packaging by seeking biodegradable packaging materials [2,3].

Paper is an excellent alternative packaging material to reduce the use of plastic and coatings. Various desirable properties of paper, such as biodegradability, recyclability, and low weight, make it a common packaging material, writing media, and household good [4]. However, its limited antibacterial and barrier properties currently represent weaknesses. These limitations pose challenges for its use in packaging materials [5]. Therefore, there is practical value in developing coating materials that enhance the physical and antibacterial properties of paper, preventing damage and bacterial infection in packaged content.

In recent years, the overuse of antibiotics has led to many bacteria becoming resistant to antibiotics. Therefore, developing methods to kill bacteria without using antibiotics or toxic compounds is crucial. A practical approach has been antimicrobial photodynamic inactivation (aPDI), which occurs through the contact between a photosensitizer and bacteria, with the photosensitizer being further activated upon exposure to light within a specific wavelength range. Upon activation, the photosensitizer generates reactive oxygen species (ROS) that cause oxidative damage to target cells and kill the bacteria. aPDI exerts strong bactericidal effects on bacteria, such as *Staphylococcus aureus* and *Escherichia coli* [6]. A significant advantage of aPDI is using environmentally friendly materials that do not cause antibiotic resistance in microbes [7]. The food industry is under increasing pressure to reduce its reliance on plastic packaging, which is derived from non-renewable resources and contributes significantly to environmental pollution and landfill waste. The CNC biopolymer coating offers a sustainable, biodegradable alternative that can reduce plastic use without compromising packaging performance.

Nanomaterials developed from cellulose have low thermal expansion rates, large specific surface areas, tensile strength, and Young’s moduli at the nanoscale [8,9,10]. Cellulose nanocrystals (CNCs) are green, environmentally friendly, inexpensive nanomaterials that can be extracted from various plants and have desirable physical and chemical properties [11]. CNCs also possess excellent nanoscale properties and are commonly used as reinforcing fillers and packaging materials in various applications [12]. Their sizeable specific surface areas, combined with the abundance of inter- and intramolecular hydrogen bonds in their internal chemical structures, enable the formation of tight structures that hinder the passage of molecules [13]. Considering the advantages above, CNCs have been hypothesized as ideal coating materials for packaging paper [14]. A plethora of hydroxyl groups exist in the chemical structure of CNCs, which facilitates their functionalization through chemical modifications to introduce other functional groups. Functionalized CNCs have several applications because they possess additional properties and can serve as carriers for other materials. One common application is in the field of bactericidal materials. Xu et al. used aldehyde-functionalized CNCs to synthesize silver nanoparticles. The dispersibility of CNCs has also been utilized to disperse silver nanoparticles in water successfully [15]. Tang et al. fabricated CNCs with antimicrobial effects by polymerizing rhodanine, which possesses antimicrobial activity, onto CNCs [16]. De Castro et al. [17] grafted rosin onto CNCs and reported that the rosin-modified CNCs exhibited antibacterial effects against Gram-negative bacteria.

Sun et al. [18] prepared a novel antibacterial poly(hexamethylene guanidine) (PHMG) cellulose material using the following process. First, glycidyl propargyl ether with epoxy groups was grafted onto azidated cellulose fibers (CFs) to obtain epoxy CFs. PHMG was then chemically grafted onto the epoxy CFs to form the antibacterial material CF-PHMG. The obtained CF-PHMG fibers exhibited long-term antibacterial effects, with 99% of the antibacterial activity retained after 60 days of storage. Sun et al. [19] prepared quaternized carboxymethyl chitosan (QCMC) and reported that the antimicrobial activity of QCMC was superior to that of CMC. Wu et al. [20] synthesized a conjugated microporous polymer (CMP)/TiO_2_ nanocomposite material by in situ Sonogashira polymerization and then demonstrated its photocatalytic antibacterial properties against *Escherichia coli* and *Staphylococcus aureus*. After 120 min of visible light irradiation, a 13-wt% CMP/TiO_2_ nanocomposite material exhibited optimal photocatalytic antibacterial rates of 98.14% and 100% against *E. coli* and *S. aureus*, respectively. The studies above indicate that combining antimicrobial functional groups with appropriate carriers can enhance antimicrobial efficiency and provide advantages such as maintaining long-term antimicrobial effects.

Jung et al. [21] utilized starch–silver nanoparticles to prepare a biodegradable coating with increased oil resistance and antibacterial activity. El-Wakil et al. [22] combined wheat gluten, CNCs, and TiO_2_ to prepare a coating film for paper, which increased the mechanical strength and water resistance of the paper and exerted antimicrobial effects through TiO_2_. Amini et al. [23] prepared a hybrid coating material by mixing cellulose nanofibers (CNFs) with silver nanoparticles and then compared their impact on kraft and greaseproof papers. The coated layer enhanced the strength of the paper by amplifying its gas barrier properties through the tight structure of the CNF. It also achieved antimicrobial effects owing to the silver nanoparticles. These examples demonstrate the feasibility of manufacturing multifunctional coated papers.

Although the abovementioned coating materials exhibited sound properties, most antimicrobial materials were fabricated using silver nanoparticles, which may pose an environmental hazard. The coating materials were prepared by directly mixing and stirring the raw materials, followed by simple adhesion onto the coating substrate. Therefore, antimicrobial agents are prone to detachment during use, which may affect the environment, human health, and service life of the packaging papers. In this study, we proposed three hypotheses to address these issues. Considering the green, innocuous, and highly effective characteristics of aPDI, innocuous erythrosine was selected as the photosensitizer [24,25], and considering the various advantages of CNCs, dialdehyde CNCs (DACNCs) were selected as carriers. Surface functionalization was achieved via periodate oxidation. The three hypotheses were as follows: (1) Functionalized cellulose can form a composite with a photosensitizer more efficiently and is less likely to cause erythrosine detachment, thereby achieving stronger and longer-lasting antimicrobial effects. (2) When packaging paper was coated with an erythrosine-DACNC (Ery-DACNC) composite, the physical properties of the multilayer film enhanced the mechanical strength and packaging performance of the paper. (3) The high-density distribution of photosensitizers on packaging paper surfaces provided excellent antimicrobial effects upon exposure to green and white lights commonly found daily.

## 2. Materials and Methods

### 2.1. Materials

The CNCs used in this study were chlorine-free and produced in our laboratory [26]. A 1-wt% CNC solution was dissolved in deionized (DI) water for sodium periodate oxidation. The solution was adjusted to a pH of 4.5 with hydrochloric acid and then heated to 45 °C. Subsequently, sodium periodate (6 g) was added to the solution and allowed to react for 12 h. After the reaction, the solution was dialyzed for 40 h and freeze-dried. The resultant products were DACNCs, which were used to prepare the Ery-DACNC composite by adding 100 mg of erythrosine to a 1-wt% DACNC solution (100 mL) and mixing it in an ultrasonic bath (. Using the molar attenuation coefficient method, 83 mg of erythrosine was confirmed to be grafted onto 100 g of DACNC. Ery-DACNC used for aPDI undergoes more than five water washes to ensure that erythrosine does not migrate to the kraft paper.

### 2.2. Ery-DACNC Composite

The coating was performed as follows: A 4.5-wt% Ery-DACNC solution was prepared and poured into a 150 mm culture dish. The kraft paper was cut into pieces with areas of 88 cm^2^. One side of each sample was soaked in the Ery-DACNCs for 5 min. The excess Ery-DACNCs were removed by washing them with distilled water for 10 s. The coated paper was air-dried, and the coating process was repeated 15 times to obtain 15 coating layers. The coating weight of the 15-layer coating is 5.7 ± 0.05 g/m^2^. The calculation method involves subtracting the weight of the paper before coating from the weight after coating and then dividing by the area of the coated paper.

### 2.3. Morphology Characterization

The surface morphology of DACNC and Ery-DACNC was observed using a JEOL JEM-2010 transmission electron microscope (TEM) (JEOL, Tokyo, Japan). VK-9500 3D profile laser microscope (Keyence, Taipei, Taiwan) was used to monitor the appearance of DACNC and Ery-DACNC powders after freeze-drying. The coating was observed using a JEOL JSM-7000 field-emission scanning electron microscope (FE–SEM) (JEOL). The bonding between erythrosine and DACNC was investigated using Nicoleti S5 Fourier transform infrared (FTIR) spectroscopy (Thermo Fisher Scientific, Madison, WI, USA). The surface morphologies of the films were analyzed.

### 2.4. aPDI Process

For the aPDI process, 20 μL of 1.5-wt% Ery-DACNCs solution was mixed with 50 μL of an *E. coli* suspension and irradiated using green- and white-light-emitting diodes (to simulate real-world lighting conditions). *E. coli* is a well-characterized bacterium that is commonly used in antibacterial research due to its clear growth characteristics, well-understood genetics, and ease of cultivation [27,28]. The effects of different irradiation times (10–30 and 60 min for green light and 20–60 and 90 min for white light) were investigated. This study utilized LED lamps from Everlight Electronic, Taiwan, which emitted two wavelengths: green light at 525 nm and white light. Each efficient light module incorporated 24 high-performing LEDs. The green LED module peaked at an irradiance of 525 nm (I40%: 500–550 nm) and delivered an intensity of 345 mW/cm^2^ over a 60.8 cm^2^ area. In contrast, the white LED module emitted a power of 650 mW/cm^2^ across the same surface area.

The antimicrobial properties of the coated papers were tested by ISO 22196-2011 standards [29]. The coated paper was cut into 2.2 × 2.2 cm^2^ samples and placed in sterile lysogeny broth agar plates. A 50 μL inoculum comprising 10^6^–10^7^ colony-forming units (CFUs)/mL was prepared and added to the coated surface of each coated paper sample. Subsequently, the samples were covered with glass coverslips and subjected to aPDI, after which the agar plates were incubated at 37 °C for 24 h. Bacterial cells were recovered with saline and plated on an agar plate for CFU counting. All tests were performed in triplicate.

### 2.5. Ery-DACNC Coating on Kraft Paper

The coating was performed as follows: A 4.5-wt% Ery-DACNC solution was prepared and poured into a 150 mm culture dish. The kraft paper was cut into pieces with areas of 88 cm^2^. One side of each piece was soaked in the Ery-DACNCs for 5 min. The excess Ery-DACNCs were removed by washing them with distilled water for 10 s. The coated paper was air-dried, and the coating process was repeated 15 times to obtain 15 coating layers. The coating weight of the 15-layer coating is 5.7 ± 0.05 g/m². The calculation method involves subtracting the weight of the paper before coating from the weight after coating and then dividing it by the area of the coated paper.

### 2.6. Water Vapor Barrier and Mechanical Properties of the Coated Paper

The water vapor barrier properties of the composite-coated paper were determined by measuring the water vapor transmission rate (WVTR) and water vapor permeability (WVP). The WVTR represents the ability of a sample to serve as a barrier against water vapor penetration per unit of time. In contrast, WVP is the amount of water vapor that passes through a unit area of a sample of unit thickness. Measurements were performed using the wet cup method (American Society for Testing and Materials (ASTM) E96/E96M-2014) [30], and calculations were performed using previously reported methods [31].

The bursting strength of the composite-coated paper was tested by the International Organization for Standardization (ISO) 2758-2014 [32] using a Mullen low-pressure-type bursting strength tester (Kumagai C, Kumagai Riki Kogyo Co. Ltd., Tokyo, Japan). This test measured the pressure at which the paper was punctured using a rubber diaphragm. The test results are reported as (1) bursting strength (kPa), which is defined as the maximum pressure that the paper can withstand before puncture, and (2) the burst index (kPa × m^2^/g), which is the bursting strength divided by the grammage of the paper.

## 3. Results and Discussion

### 3.1. Morphology Observation and Surface Bonding Analysis

**Figure 1:**
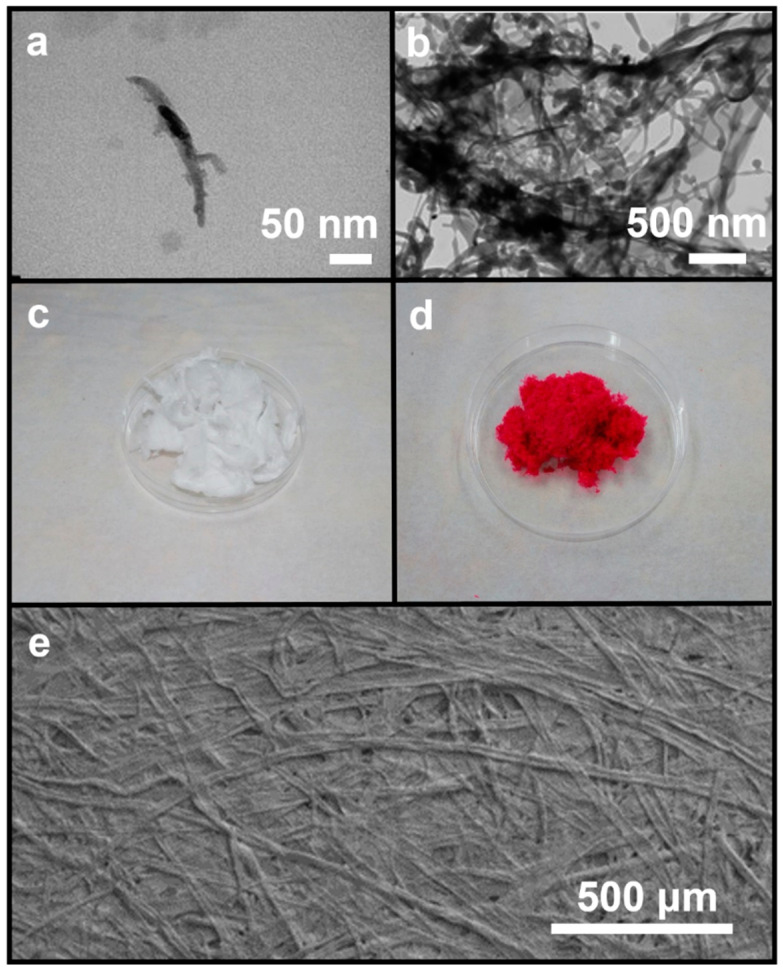
TEM image of (**a**) DACNC, (**b**) Ery-DACNC, (**c**) DACNC powder, (**d**) Ery-DACNC powder, and (**e**) SEM image of the 15-layer coated kraft paper.

Figure 1a shows the TEM image of DACNC. The appearance when compounded with erythrosine is shown in Figure 1b. Figure 1c,d show the surface morphology of DACNC powder and Ery-DACNC powder, respectively, observed under an optical microscope after oxidation and freeze-drying. Figure 1e displays the surface morphology of the Ery-DACNC coating.

**Figure 2:**
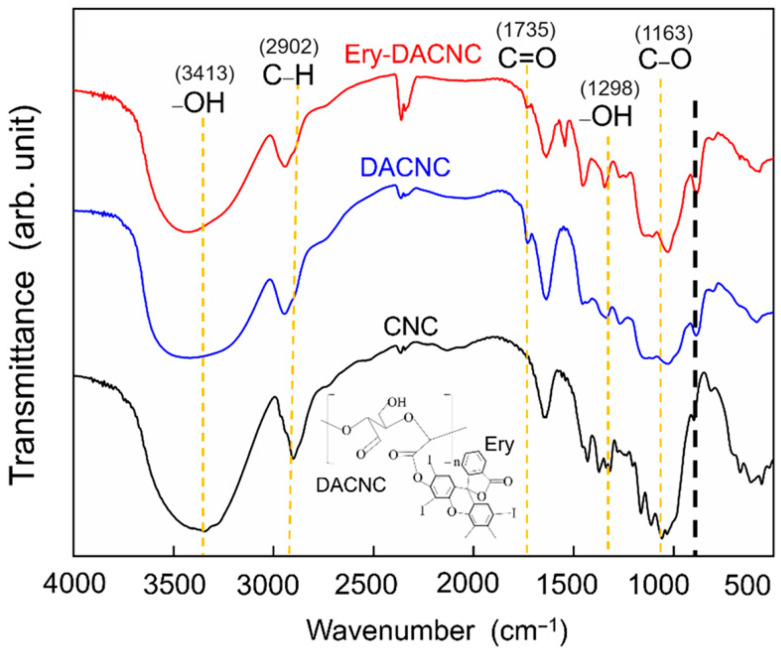
FTIR spectra of Ery-DACNC.

FTIR spectroscopy enables the investigation of the composition, structure, configuration, and changes in the molecular structures of polymer chains under certain conditions. It is also an effective method for determining the compositional distribution of polymeric materials. For example, Biswas et al. successfully identified the functional groups of cellulose and lignin using FTIR spectroscopy [33]. Figure 2 shows the results of the FTIR spectroscopy characterization. When compared to the existing literature, it validates the FTIR spectra of CNCs [34]. Both the modified and unmodified CNCs exhibited typical cellulose peaks. The broad peak between 3300 and 3500 cm^−1^ and the peak at 1298 cm^−1^ were attributed to the stretching and bending vibrations of the hydroxyl groups, respectively. The 2902 cm^−1^ and 1163 cm^−1^ peaks were attributed to the symmetric stretching vibrations of C–H and C–O bonds. Compared to the FTIR spectrum of the unmodified CNCs, the DACNCs exhibited a carbonyl absorption band at 1735 cm^−1^ [35], a red shift of the hemiacetal vibration peak at 885 cm^−1^, and a reduced intensity of -OH at 3413 cm^−1^. This demonstrates that introducing aldehyde groups into CNCs through the modification of hydroxyl groups exerts changes in the skeletal structure of the CNCs. In the FTIR spectrum of the Ery-DACNCs, binding with erythrosine caused a new carboxyl peak at 1550 cm^−1^ and the weakening of the carbonyl peak at 1735 cm^−1^, indicating that erythrosine was bound to the DACNCs through aldehyde groups. It can be seen that the intensity of the peak at 3400 cm^−1^ of DACNC and Ery-DACNC was decreased compared to CNC. This may be attributable to the presence of hydroxyl groups participating in strong hydrogen bonds.

### 3.2. aPDI Performance of Ery-DACNC Composite

**Figure 3:**
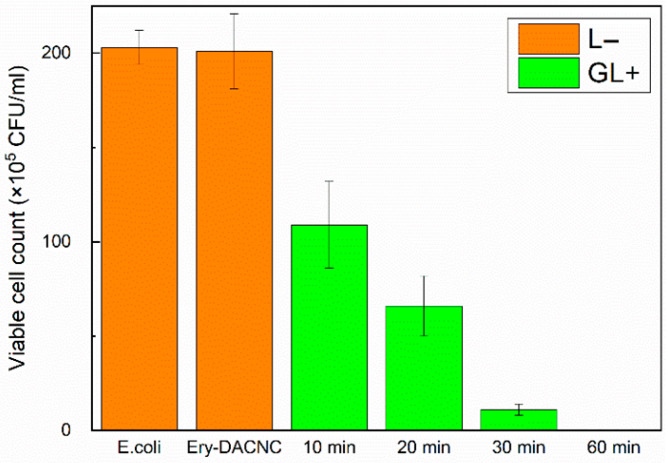
Bacterial colony counts of 1.5-wt% Ery-DACNCs under different conditions and green light irradiation.

**Figure 4:**
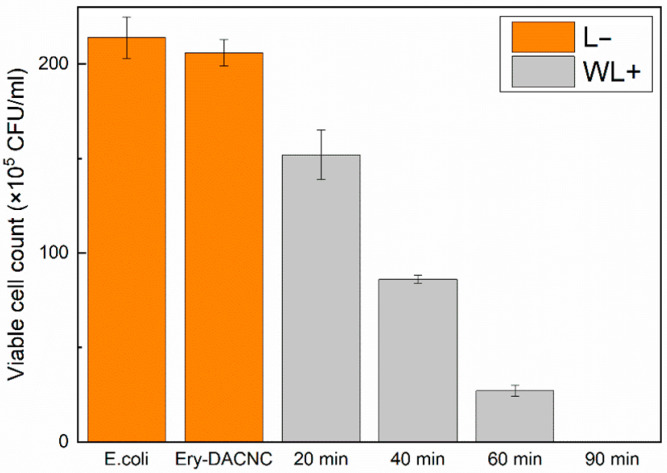
Bacterial colony counts of 1.5-wt% Ery-DACNCs under different conditions and white light irradiation.

Ery-DACNCs exhibited excellent bactericidal ability after the aPDI treatment, inactivating more than 90% of *E. coli* after 30 min of green light irradiation and 100% of *E. coli* after 60 min of green light irradiation. Figure S1a–f show the growth of bacteria in culture dishes without irradiation and under green light irradiation. Figure 3 shows the results of the CFU counting. The Ery-DACNCs exhibited lower bactericidal efficiency under white light irradiation, which simulates real-world lighting conditions, than under green light irradiation. However, they were capable of inactivating more than 85% of *E. coli* and 100% of *E. coli* after 60 min and 90 min of white light irradiation, respectively. Figure S2a–f show the growth of bacteria in culture dishes without irradiation and under white light irradiation, and Figure 4 shows the results of the CFU counting. This observation of the antibacterial properties of CNCs is consistent with, and similar to, the findings reported in previous research [36].

### 3.3. Analysis of Water Vapor Barrier and Mechanical Properties of the Coated Paper

**Figure 5:**
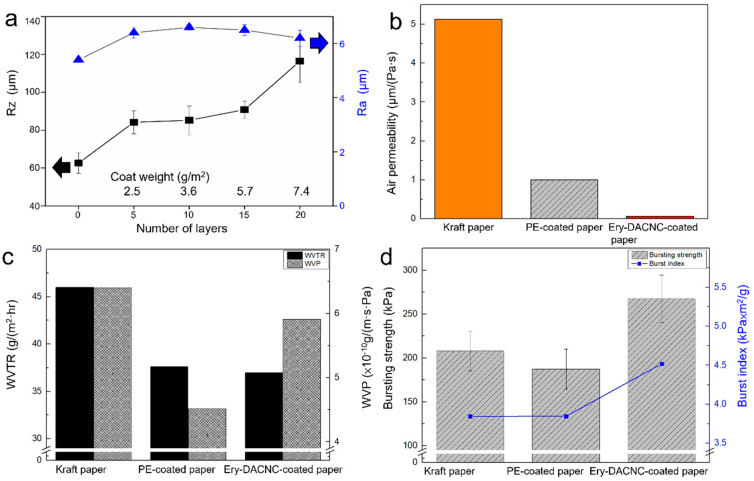
Comparison of (**a**) surface roughness, (**b**) air barrier properties, (**c**) water vapor barrier properties, and (**d**) mechanical strengths of various papers.

After a 15-layered coating, the maximum height roughness (Rz), arithmetic mean roughness (Ra), and coat weight are presented in Figure 5a. From the increasing trend in coating weight and the changes in roughness Rz, it is evident that after multiple consecutive coatings, the amount of Ery-DACNC adhered to the parchment paper increases. This, in turn, leads to an increase in the coating weight and thickness. A smooth Ra indicates that the surface remains even after coating.

Figure 5a displays the effects of a 15-layered coating on maximum height roughness (Rz), arithmetic mean roughness (Ra), and coat weight. Notably, successive layers show an evident rise in the Ery-DACNC adherence to the parchment paper, increasing coat weight and thickness. An even Ra suggests consistent surface smoothness post-coating. Figure 5b,c, respectively, showcase the air barrier and water vapor barrier properties. The WVTR and WVP define the amounts of water vapor passing through a sample under specific conditions. As the number of coatings rises, air permeability and the WVTR reduce, underscoring the improved gas barrier effect. However, growing WVP values with added layers signify a shift in the barrier effect mechanism. Figure 5d shows the mechanical strengths of Ery-DACNC-coated, uncoated, and PE-coated kraft paper. The Ery-DACNC coating, achieved through layer-by-layer application, significantly enhances the paper’s physical attributes compared to uncoated and PE-coated variants. Mainly, the bursting strength increased by 22%. The burst index increased by 15%. Air permeability dropped 72-fold. The WVTR and WVP were reduced by 24% and 8%, respectively, against uncoated kraft paper.

Ery-DACNC exhibits enhanced barrier properties against air and water vapor while strengthening kraft paper’s mechanics. Despite its advantages, the WVP of Ery-DACNC-coated paper is higher than that of PE-coated paper because of CNCs’ hydrophilic nature. The WVP of Ery-DACNC-coated paper was 42.8 × 10^−10^ g/(m.s.Pa), which is close to kraft paper’s WVP. This high WVP of CNC-coated paper is corroborated by comparisons with the literature [37].

### 3.4. aPDI Behavior of Ery-DACNC Coating

**Figure 6:**
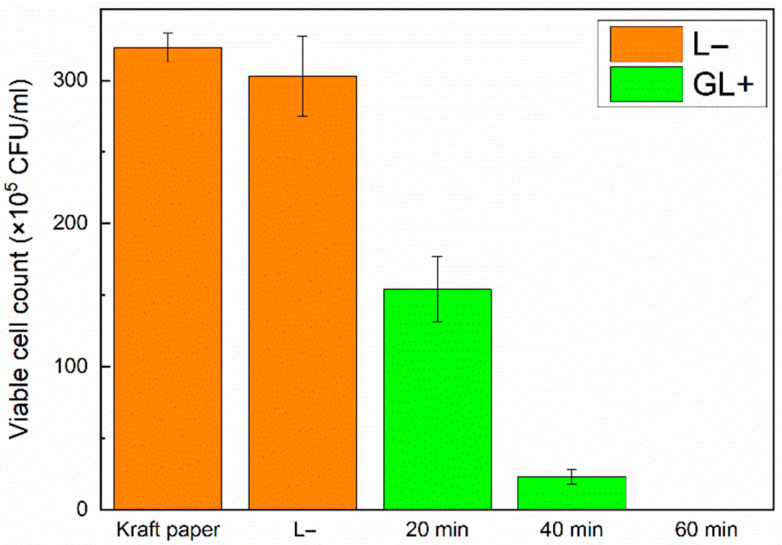
Bacterial colony counts of composite-coated papers with optimal coating parameters under different conditions and green light irradiation.

**Figure 7:**
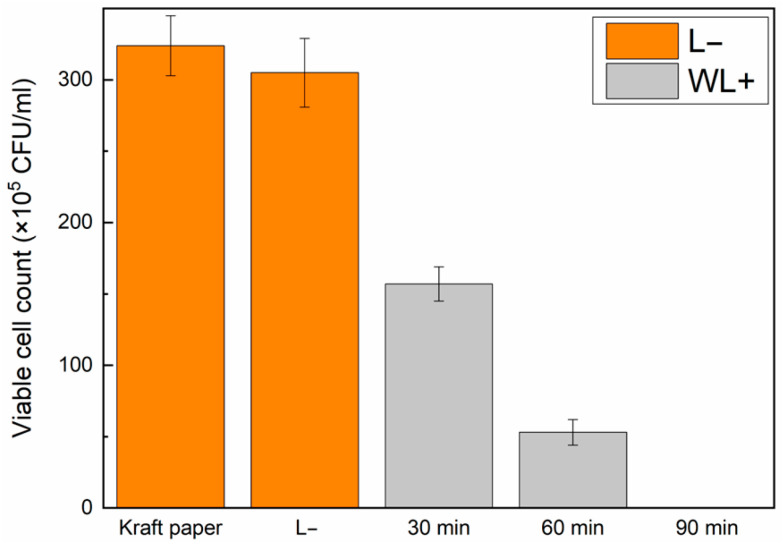
Bacterial colony counts of composite-coated papers with optimal coating parameters under different conditions and white light irradiation.

The Ery-DACNCs exhibited excellent PDI effects after being coated onto the kraft paper. The Ery-DACNC-coated paper inactivated more than 90% of *E. coli* after 40 min of green light irradiation and 100% of *E. coli* after 60 min of green light irradiation. Figure S3a–e show the growth of bacteria in culture dishes without irradiation and under green light irradiation, and Figure 6 shows the results of CFU counting. In practical applications, the Ery-DACNC-coated paper could still inactivate more than 80% of *E. coli* after 60 min of white light irradiation and 100% of *E. coli* after 90 min of white light irradiation. Figure S4a–e show the growth of bacteria in culture dishes without irradiation and under green light irradiation, and Figure 7 shows the results of the CFU counting.

## 4. Conclusions

In this study, CNCs were converted into DACNCs via periodate oxidation. DACNCs can carry an erythrosine photosensitizer to form an Ery-DACNC composite material. The photosensitizer was complexed with the open aldehyde groups on the DACNCs through intermolecular hydrogen bonding, hampering detachment. More than 80% of the added erythrosine was successfully complexed with the DACNCs, demonstrating the effectiveness and long-term usability of the composite formed between erythrosine- and aldehyde-functionalized CNCs. Neither the CNCs nor the erythrosine exerted any bactericidal effects.

In contrast, Ery-DACNCs exhibited excellent PDI activity, with more than 90% of *E. coli* inactivated after 30 min of green light irradiation and more than 85% of *E. coli* inactivated after 60 min of white light irradiation. The physical properties of the kraft paper were considerably enhanced after the layer-by-layer Ery-DACNC dip-coating process. The composite-coated paper exhibited excellent PDI effects and improved physical properties compared to the uncoated kraft paper and PE-coated paper. The Ery-DACNC-coated paper also exhibited good surface PDI activity, with more than 90% of *E. coli* inactivated after 40 min of green light irradiation and more than 80% of *E. coli* inactivated after 60 min of white light irradiation.

We achieved the aldehyde functionalization of CNCs and their complexation with erythrosine, which possesses PDI effects. Subsequently, the layer-by-layer dip-coating method was used to coat the composite material onto the kraft paper, which led to improved physical properties and conferred PDI activity to the paper. While the exact production cost of the new composite material remains limited and needs to be studied, its potential as a biodegradable and antibacterial coating for packaging paper provides a contribution to the ongoing efforts to develop sustainable alternatives to traditional plastics.

Future work will aim to address the economic analysis of this material, focusing on optimizing its production process and evaluating its cost-effectiveness in comparison with existing packaging solutions. This direction not only aligns with the global shift towards sustainable materials but also opens up new avenues for research and application in the field of environmentally friendly packaging technologies.

This suggests that the coating material can be used to reinforce packaging paper and has a high potential as a coating material for other materials. Other functional substances can be incorporated to enhance the versatility of the material for a wide range of applications.

## Data Availability

Data are contained within the article.

## Supplementary Materials

The following supporting information can be downloaded at: https://www.mdpi.com/article/10.3390/polym16070960/s1, Figure S1: Bacterial growth in 1.5-wt% Ery-DACNCs under different conditions and green light irradiation. (**a**) E.coli+, L-, (**b**) Ery-DACNC+, L-, (**c**) GL+ 10 min, (**d**) GL+ 20 min, (**e**) GL+ 30 min, (**f**) GL+ 60 min; Figure S2: Bacterial growth in 1.5-wt% Ery-DACNCs under different conditions and white light irradiation. (**a**) E.coli+, L-, (**b**) Ery-DACNC+, L-, (**c**) WL+ 20 min, (**d**) WL+ 40 min, (**e**) WL+ 60 min, (**f**) WL+ 90 min; Figure S3: Bacterial growth on composite-coated papers with optimal coating parameters and green light irradiation under different conditions. (**a**) Kraft paper+, L-, (**b**) coated paper+, L-, (**c**) GL+ 20 min, (**d**) GL+ 40 min, (**e**) GL+ 60 min; Figure S4: Bacterial growth on composite-coated papers with optimal coating parameters and white light irradiation under different conditions. (**a**) Kraft paper+, L-, (**b**) coated paper+, L-, (**c**) WL+ 30 min, (**d**) WL+ 60 min, (**e**) WL+ 90 min.
