# Supplementary material for: Erythrosine–Dialdehyde Cellulose Nanocrystal Coatings for Antibacterial Paper Packaging
Source: Polymers (Basel). 2024 Apr 1;16(7):960. doi: 10.3390/polym16070960 (PMC11013871; doi:10.3390/polym16070960)
Supplement: Supplementary file 1 [file polymers-16-00960-s001.zip › polymers-2899577-supplementary.pdf]

Supplementary Materials

Erythrosine–Dialdehyde Cellulose Nanocrystal Coatings for Antibacterial Paper Packaging

Shih-Chen Shi [1,*], Sing-Wei Ouyang [1] and Dieter Rahmadiawan [1,2]

[1] Department of Mechanical Engineering, National Cheng Kung University, No.1, University Road, Tainan 70101, Taiwan; n16074899@mail.ncku.edu.tw (S.-W.O.); n18127046@gs.ncku.edu.tw (D.R.)
[2] Department of Mechanical Engineering, Universitas Negeri Padang, Padang 25173, Sumatera Barat, Indonesia
* Correspondence: scshi@mail.ncku.edu.tw; Tel.: +886-6-2757575 (ext. 62176); Fax.: +886-6-2352-973

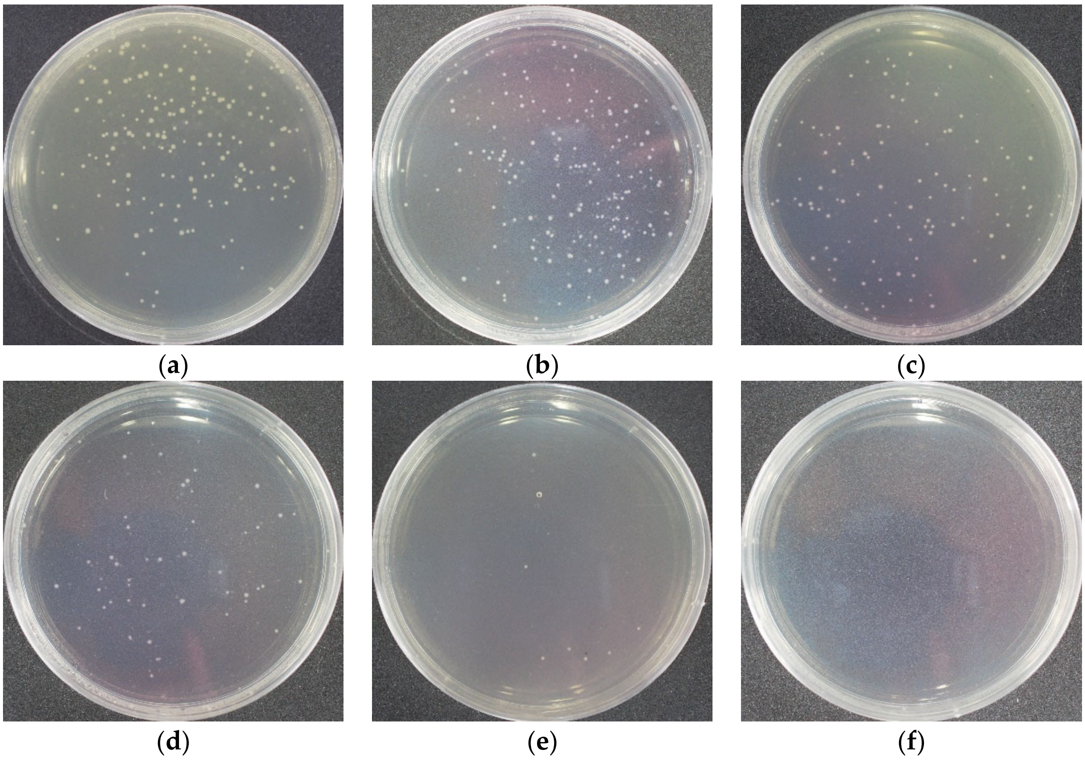

Figure S1. Bacterial growth in 1.5-wt% Ery-DACNCs under different conditions and green light irradiation. (**a**) E.coli+, L-, (**b**) Ery-DACNC+, L-, (**c**) GL+ 10 min, (**d**) GL+ 20 min, (**e**) GL+ 30 min, (**f**) GL+ 60 min.

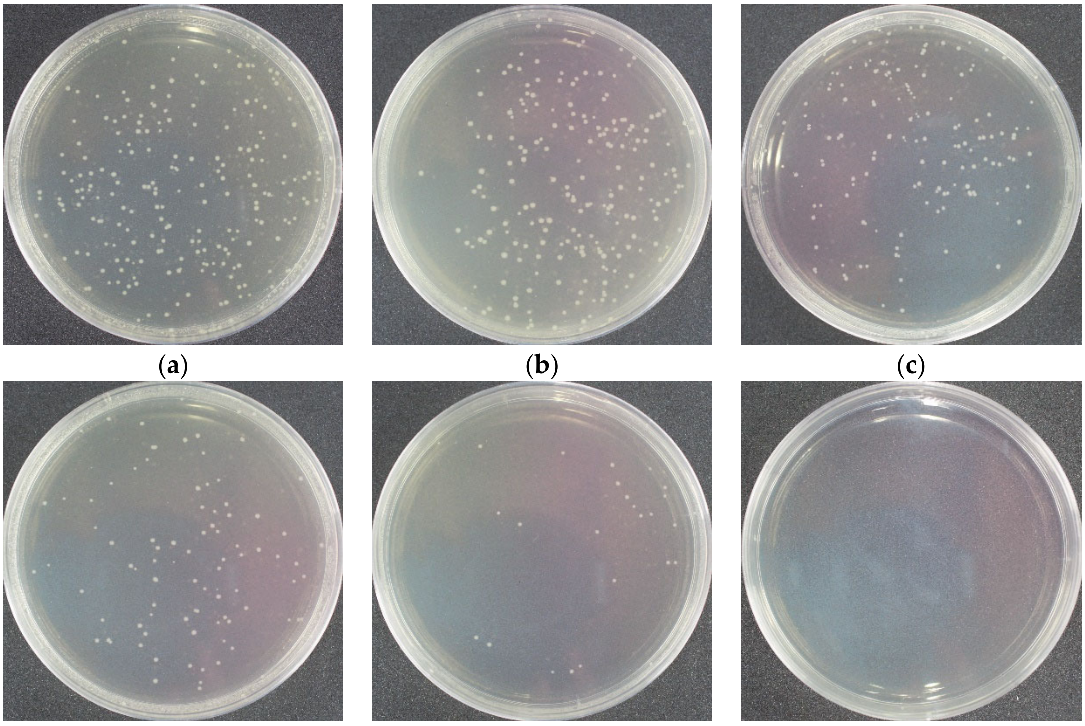

(**a**) (**b**) (**c**) (**d**) (**e**) (**f**)

Figure S2. Bacterial growth in 1.5-wt% Ery-DACNCs under different conditions and white light irradiation. (**a**) E.coli+, L-, (**b**) Ery-DACNC+, L-, (**c**) WL+ 20 min, (**d**) WL+ 40 min, (**e**) WL+ 60 min, (**f**) WL+ 90 min.

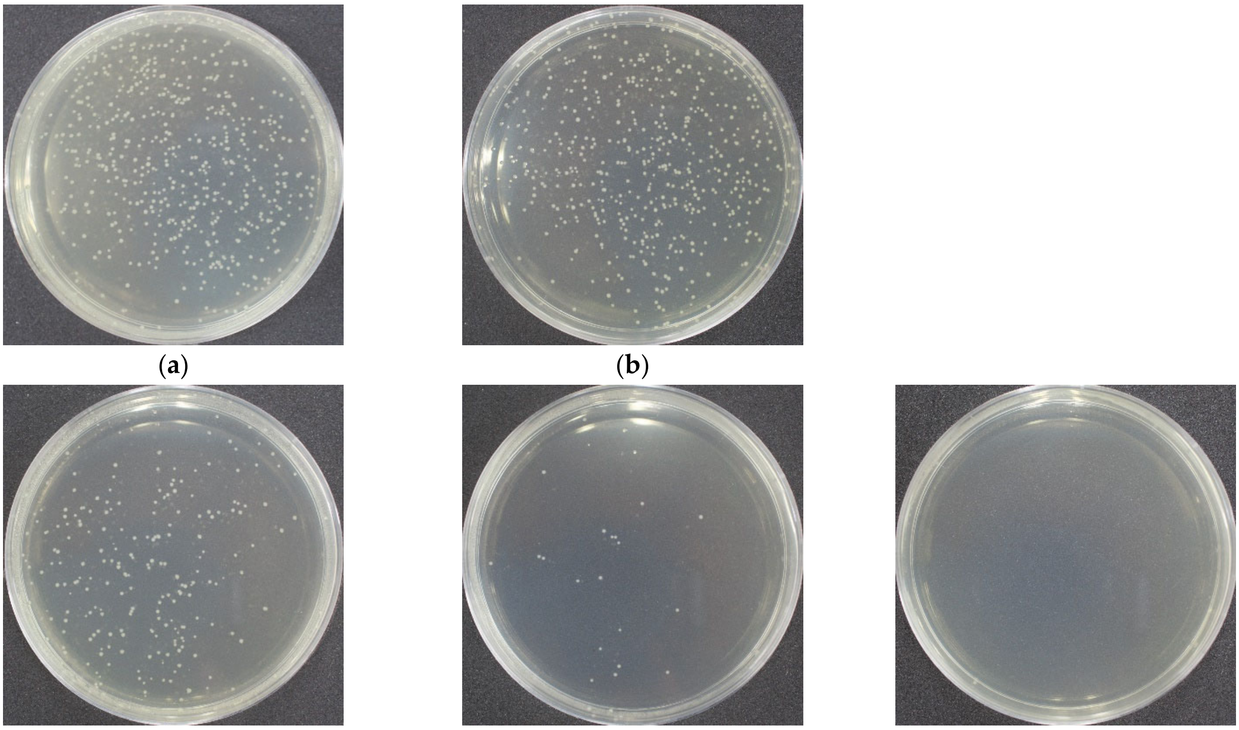

(**a**) (**b**) (**c**) (**d**) (**e**)

Figure S3. Bacterial growth on composite-coated papers with optimal coating parameters and green light irradiation under different conditions. (**a**) Kraft paper+, L-, (**b**) coated paper+, L-, (**c**) GL+ 20 min, (**d**) GL+ 40 min, (**e**) GL+ 60 min.

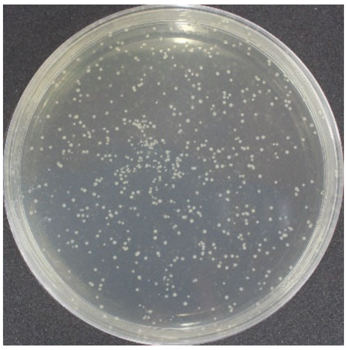

(**a**)

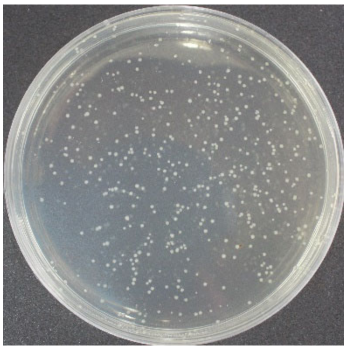

(**b**)

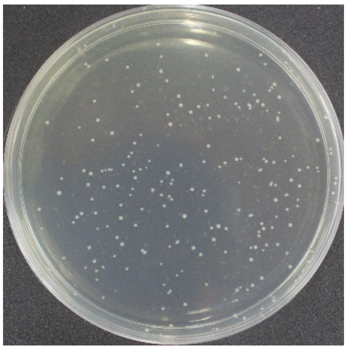

(**c**)

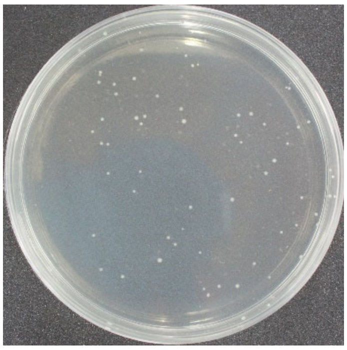

(**d**)

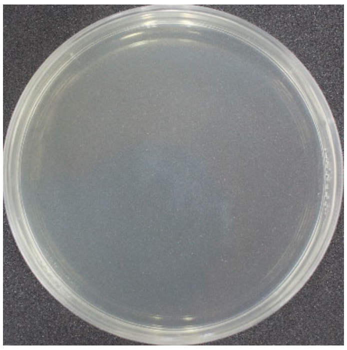

(**e**)

Figure S4. Bacterial growth on composite-coated papers with optimal coating parameters and white light irradiation under different conditions. (**a**) Kraft paper+, L-, (**b**) coated paper+, L-, (**c**) WL+ 30 min, (**d**) WL+ 60 min, (**e**) WL+ 90 min.
